# A descriptive report of management strategies used by chiropractors, as reviewed by a single independent chiropractic consultant in the Australian workers compensation system

**DOI:** 10.1186/1746-1340-17-12

**Published:** 2009-11-18

**Authors:** Henry Pollard, Katie de Luca

**Affiliations:** 1 Adjunct Professor, School of Medicine, University of Notre Dame, Sydney, Australia; 2 Macquarie Injury Management Group (MIMG), Department of Health & Chiropractic, Macquarie University, Sydney, Australia

## Abstract

**Background:**

In New South Wales, Australia, an injured worker enters the workers compensation system with the case often managed by a pre-determined insurer. The goal of the treating practitioner is to facilitate the claimant to return to suitable duties and progress to their pre-injury status, job and quality of life. Currently, there is very little documentation on the management of injured workers by chiropractors in the Australian healthcare setting. This study aims to examine treatment protocols and recommendations given to chiropractic practitioners by one independent chiropractic reviewer in the state of New South Wales, and to discuss management strategies recommended for the injured worker.

**Methods:**

A total of 146 consecutive Independent Chiropractic Consultant reports were collated into a database. Pain information and management recommendations made by the Independent Chiropractic Consultant were tabulated and analysed for trends. The data formulated from the reports is purely descriptive in nature.

**Results:**

The Independent Chiropractic Consultant determined the current treatment plan to be "reasonable" (80.1%) or "unreasonable" (23.6%). The consultant recommended to "phase out" treatment in 74.6% of cases, with an average of six remaining treatments. In eight cases treatment was unreasonable with no further treatment; in five cases treatment was reasonable with no further treatment. In 78.6% of cases, injured workers were to be discharged from treatment and 21.4% were to be reassessed for the need of a further treatment plan. Additional recommendations for treatment included an active care program (95.2%), general fitness program (77.4%), flexibility/range of movement exercises (54.1%), referral to a chronic pain specialist (50.7%) and work hardening program (22.6%).

**Conclusion:**

It is essential chiropractic practitioners perform 'reasonably necessary treatment' to reduce dependency on passive treatment, increase compliance to active care programs and reduce the progression to chronic pain states. It is recommended that common findings be integrated in further research, to improve the management of treatment for patients with an occupational injury.

## Background

Literature supports the use of chiropractic management for acute and chronic presentations of low back pain [1,2], neck pain [3,4] and extremity conditions [5,6]. Cases in which pain exists for longer than three months is termed chronic pain and it is understood that chronic pain has a greater risk for progressive pain and dysfunction [7], particularly in the workers compensation setting [8]. Risk factors for chronic pain include socioeconomic status, race, working environment, education and emotional status [9,10]. These are amongst psychosocial variables that are referred to as "yellow flags" and these variables complicate the prognosis for the chronic pain patient (Table 1) [11,12]. Of particular interest is the prognosis for people injured whilst at work. In Australia, a claimant enters the workers compensation system and their case is often managed by a pre-determined insurer [13]. An important goal of the practitioner (in conjunction with an occupational rehabilitation provider and claims officer representing the insurer) is to develop a return to work program and facilitate the claimant to return to suitable duties and progression to their pre-injury status and quality of life.

**Table 1:** Yellow flags: Psychosocial factors which may contribute to long-term distress, disability and chronic pain.

Factors important in predicting poor outcomes:
• Belief that pain is harmful or disabling
• Fear-avoidance behaviour and reduced activity
• Tendency to low mood and social withdrawal
• Dependence on passive treatment rather than active participation

In many jurisdictions, chiropractors act as primary contact allied health professionals in the workers compensation system [14-16]. In New South Wales they can render eight treatments prior to seeking approval to continue care [17]. In this setting, chiropractic management may take many forms; however, it is important that the scope and provision of treatment conforms to evidence-based management of chronic pain [18]. Inherent in this acceptance is the application and integration of active therapy [19] and other healthcare approaches through a team-based management approach [20,21]. Multi-modal management (MMM) is defined as the combination of manipulative therapy with exercise, stretching, soft tissue therapy, active care programs and other ancillary therapies. MMM of the spine [18] and extremities [5,6] is documented. Exercise rehabilitation protocols are also an effective treatment for pain and dysfunction in mechanical neck disorders [22], and other reviews have determined that manipulation and/or mobilisation results in superior outcomes when accompanied by exercise [23].

The chiropractic paradigm of "maintenance care" is defined here as the provision of manipulative therapy for the prevention of pain, dysfunction and the maximisation of health potential. It is an approach preferred by many chiropractors [24]. In this report "pre-injury status" is defined as the ability to perform work duties with the same degree of function prior to the work related injury. As defined, "pre-injury status" also infers work status is equal to that of both pre-injury duties and hours of employment" [17]. "Reasonably necessary treatment" is defined in Table 2[17]. The understanding of this term sometimes causes conflict between insurer representatives and practitioners. In some cases, treatment may continue for many years in the attempt to resolve issues associated with chronic cases by addressing "maintenance" or "wellness" factors irrelevant to the definitions of pre-injury status that are important to the insurer and the workers compensation system. It should be noted that "maintenance" or "wellness" care is precluded under the New South Wales Workers Compensation system and this is made clear to the Independent Chiropractic Consultant upon their commencement.

**Table 2:** The definition of "reasonably necessary treatment".

"Reasonably Necessary Treatment"
• "Appropriateness" of treatment
• Availability of alternative treatments
• Cost of treatment
• Effectiveness (actual or potential) of treatment
**In which "appropriate" treatment must**:
• Lessen the effects of injury
• Cure the injury
• Alleviate the symptoms of injury
• Retard progressive deterioration

### The Independent Chiropractic Consultant

An Independent Chiropractic Consultant (ICC) is appointed by the Worker Compensation Authority in the state of NSW, Australia (WorkCover NSW). The appointment follows an application and then panel interview of profession and industry members. The ICC functions independent to the insurer and practitioner and can not render treatment as a part of the consultative process. The ICC is contacted by an insurer to perform a review of the management of a claimant currently seeing a chiropractor for treatment of an occupational injury. Upon contact, the ICC is informed of the type of review required. There are three types of reviews and these are referred to as stage 1,2 or 3 reviews. A stage 1 review involves the examination of insurer files only, whilst a stage 2 review involves a review of files plus a telephone interview of the treating chiropractor discussing all aspects of assessment and management. A stage 3 review requires the ICC to review files and to contact the treating practitioner to discuss the current treatment after the ICC has conducted a consultation and examination of the injured worker. A report is generated for each of these interventions. Stage 1 reviews have been discontinued as insurer files typically did not provide useful representation of the treatment, goals and motivations of the practitioners. This study focuses on reports generated from stage 2 and stage 3 reviews.

A central requirement of each review is to determine if "pre-injury status" has occurred and whether the treatment being rendered is considered "reasonably necessary treatment". Any decision taken occurs at the discretion of the consultant after orientation and training by WorkCover. In this role, the consultant is expected to make recommendations after negotiating with the practitioner based on current best practice in the field. Where possible, it is hoped that the practitioner will agree to the recommendations after they have been explained and that agreement is noted in the subsequent report. It is noteworthy that recommendations should be made with the support of a body of peer-reviewed evidence.

Reviews by an ICC in the compensation system aim to combine scientific evidence and clinical experience to assist the clinical decision making process used by practitioners in recalcitrant cases. Focus is not only given solely to treatment type (such as technique type) but whether the treatment is successful, reasonable in its applications and is aimed at improving the worker's functional status and capacity to work.

Currently, there is very little documentation on the management of injured workers by chiropractors in the Australian healthcare setting. This study aims to examine treatment protocols and recommendations given to chiropractic practitioners by one independent chiropractic consultant in the New South Wales Workers' Compensation system, discuss the management strategies recommended for the injured workers and make recommendations for chiropractors working in the compensation system. It is important to note that the opinions expressed in this report are those of the authors and not WorkCover NSW, or any insurer, practitioner or patient described herein.

## Methods

### Analysis of the ICC report

Consecutive stage 2 and stage 3 reports conducted by one ICC in Sydney Australia were retrospectively analysed. This consultant reviewed claimants' primarily from the main population centres of the Sydney, Newcastle and Wollongong regions of New South Wales. All personal identifying information of the injured workers and practitioners was omitted from the database. Data tabulated and analysed for trend included the type of management, how it had changed over time and whether management would change in the future; the history of the injury such as the location, severity, duration, aggravating and relieving factors; and other treatment variables such as medical history and biopsychosocial variables. The data formulated from the reports are purely descriptive in nature. Recommendations made by the ICC to the treating practitioners were also tabulated and analysed for trend.

### Outcome Measures

The Chiropractors' Guide to WorkCover NSW states that outcome measures of pain and disability should be utilised by all practitioners when managing patients injured in the workplace. For a copy of this guide see the WorkCover website at: http://www.workcover.nsw.gov.au/ServiceProviders/HealthCare/Pages/Chiro.aspx. These measures assist in quantifying the level of pain and disability as well as the effectiveness of therapy. When used as a primary goal of treatment, these measures provide clinical justification for the use for effective interventions. Two main outcomes are "work status" and "functional restrictions". They provide focused goals for returning the injured worker to the workplace.

## Results

A total of 146 consecutive ICC reports were generated from the 10^th ^of January 2005 until the 21^st ^of November 2006. Of these reports, 44.5% were Stage 2 reviews and 53.4% were Stage 3 reviews. Some data was missing from reports where practitioners could not report it from their injured worker records, however much of this was not relevant to the findings of this review.

### Injured Worker Demographics

We found that 58.2% of the injured workers were male and 41.8% were female. The injured worker cases ranged from acute stage cases (up to three months), to long term cases (greater than 10 years of consecutive compensation), with the average duration of the compensation claim to be 5.2 years (SD = 4.3 yrs). All but one of the cases was chronic in nature with most cases being more than two years in duration. Due to the case mix, the nature of the recommendations herein contained relate to the chiropractic management of chronic pain states. In 45.9% of cases the primary complaint was low back or lumbosacral pain, whilst 37.0% reported a cervico-thoracic complaint. Statistics showed that 41.8% of injured workers reported pain waking or interrupting their sleep, 54.1% were on some form of medication for their pain, 31.5% of the injured workers had been involved in a motor vehicle accident and 41.1% of injured workers had some form of pre-existing injury to the region being treated under compensation. Imaging studies (x-ray, CT, MRI or bone scans) were performed on 89.0% of the injured workers, with many having multiple images that were serially performed (most frequently ordered by their nominated treating medical doctor or for documentation in medicolegal cases).

### Findings of the ICC report

Prior to the current chiropractic care, 72.6% of injured workers had some form of other treatment. Significantly, 73.6% had had their previous treatment in the form of physiotherapy. In 67.1% of the cases, injured workers reported some form of psychosocial issue. Of these, 49.0% demonstrated a dependency on passive and 17.3% appeared to demonstrate fear avoidance behaviour as discussed in the interview. Noteworthy were 18.4% of injured workers whom reported suffering from stress directly related to the insurers' management of the case. In many cases, more than one psychosocial variable was reported. Recommendations for such cases were to be referred to an appropriate practitioner for integrating psychosocial and behavioural interventions as recommended by current management guidelines [17]. Despite these guidelines, much research is still required to conclusively validate the need for such approaches [25].

The scheduling of treatment at the time of the review ranged between three times per week to once every six months. The consultant determined the current treatment plan to be "reasonable" in 80.1%, and "unreasonable" in 23.6% of the cases. In eight cases treatment was unreasonable and immediate cessation of treatment was recommended, whilst in five cases the treatment plan was deemed reasonable and treatment was discharged. In these cases treatment was discharged because the claimant had reached pre-injury status. Of 117 cases in which treatment was reasonable, 74.6% of practitioners were recommended to "phase out" treatment. The ICC recommended that 78.6% of the injured workers were to be discharged at the end of the scheduled treatment, whilst 21.4% were to be reassessed for the need of further treatment. A mean number of visits 8.4 visits (SD = ± 4.6 visits) to the treating practitioner were recommended for the injured worker before being discharged from further treatment.

### Recommendations made by the ICC

The consultant recommended various management strategies to be incorporated into the injured worker's management program. These recommendations were negotiated with the practitioner and agreement or disagreement with the protocol was noted in the ensuing report. Only a small number of practitioners disagreed with the recommended protocol and the disagreement generally centred on a conflict of philosophical approaches to treatment or a lack of understanding that the goal of management was for the return to "pre-injury status" and not the complete absence of pain or for "maintenance" therapy. An arbitrary rating scale from 0 to 100 (where whilst 0 reflects a total inability to perform any pre-injury duties and 100 is complete ability to perform pre-injury duties) was used to rate the injured worker's perception of return to function. The average the pre-injury status of an injured worker was 72.7% (SD = ± 21.4). The recommendations rarely required additional manual therapy but frequently required the addition of other forms of therapy. All recommendations made by the ICC can be found in Table 3.

**Table 3:** Recommendations made by the Independent Chiropractic Consultant to the treating practitioner for inclusion in the claimants' chiropractic management program.

Recommendations for inclusion in the Chiropractic management program		
	***n***	**%**
Active therapy program	139	95.2
General fitness program	113	77.4
Flexibility and range of motion exercises	79	54.1
Referral to a chronic pain specialist (a psychologist or psychiatrist with a cognitive or behavioural approach)	74	50.7
Work hardening program	33	22.6
Referral to a physiotherapist	21	14.4
Dietary consultation	11	7.5
Surgical intervention	6	4.1
Post-surgical rehabilitation	1	0.7
Neurological consultation	1	0.7
Other: Understanding of "reasonably necessary treatment", back support, ergonomic evaluation, workplace assessment, assault management, utilising outcome measures, job placement advice, re-evaluation of medications and referral to a podiatrist	82	56.2

Recommendations made by the ICC were made on the basis that management should contain active and passive components and that the condition should be improving. If this was the case, no remedial action was recommended. If pain was static as was the case in the majority of cases, the role of active therapy, psychosocial variables or whether change had occurred in the delivery of the passive therapies was discussed and or recommended. If the management strategies appeared to be governed by a philosophical approach that was not consistent with a return to pre injury status governed by reasonably necessary treatment, a reduction, change or cessation of care was recommended. Where possible, research material or the Workers' Compensation Act of NSW was used to reinforce the concepts being discussed. When all of the above had been reasonably implemented but the case could still not be resolved (a small number of the total), the injured worker was referred to a medical or other healthcare specialist for review.

## Discussion

This paper presents a review of 146 consecutive ICC reports that examined the treatment protocols of, and recommendations to, treating practitioners and the injured workers. The pursuit of patient centred, evidence-based care should be the goal of all chiropractors. In addition to such management goals is the need to address Workers Compensation claims in a timely and effective manner. However, in some cases efficient return of the injured worker to pre-injury status is not achieved. There are many potential reasons for this problem, which include difficult cases, multi-region pain syndromes, recurrent injury, lack of change in approach to treatment regardless of stage of management, lack of recognition of psychosocial variables, lack of active therapy, lack of co-management, pursuit of wellness or maintenance care approaches, lack of understanding of the definition of reasonably necessary care under the workers' compensation system in NSW and a lack of recognition of the need to cease treatment once the pre-injury status had been achieved.

It is widely accepted that after three months an injury is deemed chronic and whilst chiropractors are recognised as effectively treating chronic pain, management by practitioners for long periods of time in the absence of any improvement or after the pre injury state has been reached possibly questions the focus of the practitioner [26]. We found the scheduling of treatment ranged from three visits per week, to two visits in 15 months, demonstrating a wide spectrum of scheduling protocols for injured workers that were not always consistent with the attainment of the pre injury status. Injured workers are subjected to an intervention driven by the philosophical paradigm of the chiropractor. Maintenance management highlights the need to educate the patient in a holistic way, using traditional epistemologies of wellness and elevated patient health for long-term management [27]. Whilst this may be appropriate in supporting the responsibility of self-health for the purpose of maximising one's own self funded health potential, the same goal is by definition inappropriate in the workers compensation setting.

In further discussion of the need for clear and defensible management guidelines, we found a frequent misunderstanding of the term "reasonably necessary treatment" (Table 1) by both the practitioner and the injured worker. It is our experience that this misunderstanding often stems from a misinterpretation of the terms of court settlements and remains a strong motivating factor for receiving ongoing care in our opinion. A frequent recommendation is that the term "reasonably necessary treatment" is defined clearly for the claimant by the insurer or the legal representative of the claimant. Due to the frequency at which this misunderstanding seems to occur we further recommend that legal representatives clearly define this term so that claimants do not form the opinion that they have won a court ruling that entitles them to treatment indefinitely.

Chiropractic management must aim to return the worker to pre-injury status, in an efficient and effective manner. This often means a multi-modal approach should be considered [28]. Such management often incorporates the pursuit of pain reduction and functional restoration by a variety of methods by physical, occupational, pharmacological, psychological, behavioural, and surgical amongst others [29]. With literature providing evidence for multi-modal management of work related disorders [30], the possibility exists that at a time not too distant from today when more evidence for such approaches will be available, that the treating practitioner may be at risk of not only losing insurer support for treatment protocols, but they may be liable for litigation (by insurer or claimant) for not providing "reasonably necessary treatment".

The ICC recommended forms of therapy for inclusion into the chiropractic management that are designed to increase the effectiveness of returning the injured worker to pre-injury status. The results can be found in Table 3. Recommendations are made for various reasons. The most common reason for an intervention appears to be because management lacks direction following a plateau of outcomes. Another common reason for intervention includes those cases where management outcomes seem more appropriate for acute interventions rather than for more chronic presentations.

In nearly all of the ICC reports it was recommended that the injured worker be engaged in an active therapy program, and in a majority of reports it was recommended that a general fitness program and flexibility/range of motion exercises be performed for effective management. This is consistent with the literature on chronic pain management [19,31,32]. In particular, evidence exists that treatments that are active rather than passive are associated with better outcomes [33]. Active therapy is imposed to motivate individuals to independently control their functional wellbeing and administer safe, effective, relevant and uncomplicated exercise programs to enhance the rehabilitation regime [34,35].

Noteworthy to this study, we found that 67% of the injured workers reported some form of psychosocial "issue". The "issue" was identified by the ICC as one that became apparent in the consultation or examination. These issues included a suspicion based on the New Zealand Acute Low Back Pain Guide [11]. A significant finding was that 40% of injured workers were "dependant on passive therapies". Dependence is known to occur with long term passive therapy management, and highlights the responsibility of the practitioner to return the injured worker to pre-injury status as soon as practical. Whilst management that incorporates active therapy is appropriate, it is the inappropriate application of the wellness paradigm to occupational chronic pain which may perpetuate the dependence on passive therapy and prolong rehabilitation [36]. It is possibly this philosophical approach that has previously shown chiropractors to retain patients in a non work setting longer than their physiotherapy or osteopathic colleagues [37].

Based on this report, many practitioners assist in rehabilitation whilst others do not. Various reasons are given. The most common approach is one where exercises are given verbally or on a sheet of paper and then never followed-up. Another group sparingly monitors prescribed exercises and yet another group deem the provision of exercises to be the domain of other health care providers. The latter approach highlights an older chiropractic philosophical approach to management that is driven by the provision of manipulative therapy as a monotherapy rather than as a therapy that is a component part of a multimodal approach to management preferred by many [5,6].

It seems apparent that there is a need for a change of attitude in some practitioners and injured workers, and a need to embrace active based care [38]. The statutory authorities could assist this process with continuing educating campaigns directed to both claimants (via claims officers) and practitioners, which would include disseminating information on best practices for managing barriers and facilitating return to work. Whilst not in the scope of this review, it should also be noted that an employers willingness and ability to facility the injured worker to return to work is crucial in good outcomes. Employers too should be included in education campaigns and best return to work practices, whether it is restricted hours, duties, job placement or identifying and minimising barriers to return to work.

Research clearly shows that education of an injured worker is a desirable pursuit [39]. However, broad based public health campaigns whilst thought initially to benefit society [40,41], have recently come into question as a viable means of reducing worker disability [42]. Injured workers' should be educated as to the effect and likely progression of an injury, what is likely to help and hinder and what to expect in terms of exacerbations and remissions. Furthermore, they should be instructed to employ a raft of self-management and coping strategies to manage pain, and also rehabilitate themselves through compliance to exercise programs. Collectively, these measures attempt to instil a sense of self- responsibility for the rehabilitation of their injury [43,44].

"Fear avoidance" was another commonly described issue with an injured worker. The literature reports such characteristics in chronic pain cases and it should be assessed by practitioners and specifically managed [45]. Feelings of frustration, anxiety, stress and "I want my life back" and/or "I will never get better" statements were commonly reported by the injured workers. These feeling are complicated by confusion associated with the wellness paradigm as practitioners tell their patients that they will always need treatment (maintenance). The problem lies in the miscommunication of a pain and disability construct (by the patient) with one of health promotion/performance (by the practitioner). Despite the maintenance being rendered under a different treatment paradigm, a strong potential for confusion exists in susceptible individuals. Further research should investigate these outcomes. The relevance of the adoption of a biopsychosocial model of management by chiropractors has previously been discussed [46], and supports reassurance by the chiropractor as an important part of the practitioner interaction [47]. It is important that a good working understanding of "yellow flags" [11] and their recognition, assessment, and management implications for chiropractors operating in the workers compensation system is essential for the well-being and effective recovery of the injured worker [48].

The findings of this study highlight various management strategies for the effective management of injured workers and some possible pitfalls. For any chiropractor managing injured workers in the workers compensation system it is imperative that management protocols and record keeping have defensible and definable management outcomes that adhere to accepted evidence-based guidelines about returning the injured worker to work [49,50]. The use of published guidelines based on best evidence syntheses is important for all primary healthcare practitioners. Failure to do so has been associated with poor outcomes [51]. Unfortunately, there is evidence that primary healthcare practitioners are not keeping up to date with published guidelines and this is true of management of occupational low back pain in Australia [52]. This report provides indirect evidence to support that a minority of chiropractors are also limited in their application of evidence based guidelines. However, the application of guidelines alone may be insufficient in the absence of truly patient centred care [53]. The consideration of reasons why guidelines are not being considered is beyond the scope of this report although it has been suggested that the contradictory nature of the guidelines between various professional groups may be barriers to adherence [54]. Inherent in this process is the acquisition of "pre-injury status" and the limitation of treatment to that which is considered "reasonably necessary" by WorkCover guidelines regardless of other non-work related management paradigms.

### Limitations

This study analysed data generated from the reports of *one *ICC. Therefore, whilst the recommendations given are evidence based in nature, recommendations given are based on the chiropractic management paradigm of this one consultant. As a result, the recommendations may not be consistent with others within the same system or elsewhere. In addition, recommendations may or may not have been multi-modal in nature. Furthermore, the authors only reported specific recommendations made to the treating practitioner at the time of the review and not other underlying assumptions of clinical management.

Reports were generated in consultation with the current treating practitioner (a chiropractor). Many injured workers' had a past and or current history of multiple practitioner interventions since the time of initial complaint. This included treatment from general practitioners, physiotherapists, psychologists, other chiropractors, massage therapists and surgical interventions. Whilst due recognition of the other activities was noted, the recommendations were specifically about the chiropractic intervention and how it could (if possible) be progressed.

## Conclusion

This study reviewed chiropractic management protocols and recommendations given to chiropractic practitioners by one Independent Chiropractic Consultant as a part of an insurer quality control process. It descriptively reports the recommendations, which includes the continuation, modification or cessation of chiropractic treatment. The most common recommendation of the ICC was modification of care to include various integrated active therapy strategies that were limited to a fixed number of ongoing sessions.

It is essential chiropractic practitioners preform 'reasonably necessary treatment' to reduce dependency on passive treatment, increase compliancy to active care programs and reduce progression to chronic pain states. It is recommended that common findings be integrated in further research, which should aim to improve the management of patients with an occupational injury.

## Competing interests

HP is an Independent Chiropractic Consultant to the WorkCover Authority of NSW.

## Authors' contributions

HP: Conceived the design of the study and drafted and edited the manuscript.

KD: Participated in the design of the study, conducted the retrieval and analysis of data and drafted the manuscript.

All authors read and approved the final manuscript.
